# Spleen Area Affects the Performance of the Platelet Count–Based Non-invasive Tools in Predicting First Hepatic Decompensation in Metabolic Dysfunction–Associated Steatotic Liver Disease Cirrhosis

**DOI:** 10.1016/j.jceh.2025.102596

**Published:** 2025-05-27

**Authors:** Marcello Dallio, Mario Romeo, Fiammetta Di Nardo, Carmine Napolitano, Paolo Vaia, Simone Olivieri, Marco Niosi, Alessandro Federico

**Affiliations:** Hepatogastroenterology Division, Department of Precision Medicine, University of Campania Luigi Vanvitelli, Piazza Miraglia 2, 80138, Naples, Italy

**Keywords:** ultrasound, liver cirrhosis, liver related events, spleen area, predictive model

## Abstract

**Background/Aims:**

Various non-invasive tools (NITs) predicting first hepatic decompensation (HD) in advanced chronic liver disease (ACLD) enclose platelet (PLT) count. A relevant proportion of metabolic dysfunction–associated steatotic liver disease (MASLD)-ACLD patients do not show splenomegaly- and hypersplenism-related thrombocytopenia. We aimed to evaluate the performance of NITs in predicting HD according to ultrasound-assessed spleen size.

**Methods:**

In this observational study, 148 splenic and 27 asplenic (ASP) MASLD-compensated advanced chronic liver disease (cACLD) patients were enrolled. Ultrasound artificial intelligence–based tools distinguished splenomegaly-affected patients (SAPs) and normal-spleen patients (NSPs). Albumin-Bilirubin score (ALBI) and PLT count–based NITs (PLNs) (Fbrosis-4 [FIB-4], ALBI-FIB-4, red cell distribution width-to-PLT ratio [RPR], liver stiffness measurement [LSM]-to-platelet ratio [LSM/PLTr], and ANTICIPATE ± non-alcoholic steatohepatitis [NASH]) were determined. Over 3 years, the first HD was recorded.

**Results:**

Limitedly to SAP, spleen area inversely correlated with PLT (relationship [R]: −0.981; *P* < 0.0001), confirming the role of splenomegaly-related hypersplenism in conditioning thrombocytopenia. HD occurred similarly in SAPs (20.48%), NSPs (21.15%), and ASP patients (25%) (*P*: 0.198). In NSP, PLNs showed a reduced influence on HD (FIB-4 [*P*: 0.03], ALBI-FIB-4 [*P*: 0.001], RPR [*P*: 0.002], LSM/PLTr [*P*: 0.01], and ANTICIPATE ± NASH [*P*: 0.001]) compared to SAP. In NSP, the spleen area was inversely associated (adjusted sub-distribution hazard ratio: 0.870) and more significantly (*P* < 0.0001) impacted HD. Consistently, unlike SAPs, in NSPs and ASP patients, PLNs showed poor performance, and exclusively ALBI maintained a good accuracy (NSP: area under the curve [AUC]: 0.651, *P*: 0.04; ASP patients: AUC: 0.625, *P*: 0.03) in predicting 3-year HD.

**Conclusion:**

Ultrasound-assessed spleen size affects the predictive performance of the PLNs in MASLD-cACLD patients.

## Graphical abstract

**Figure:**
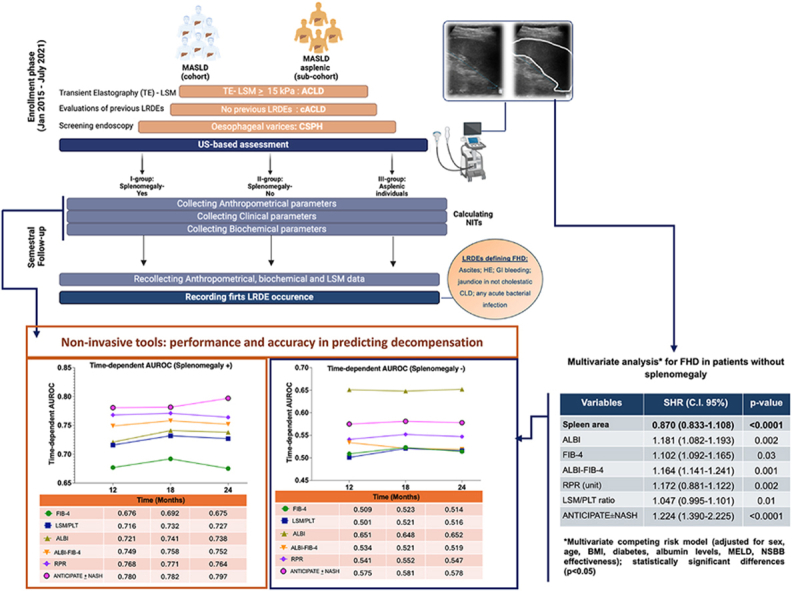

In compensated advanced chronic liver disease (cACLD) patients, clinically significant portal hypertension (CSPH), promoting liver-related decompensation events (LRDEs),^1^ constitutes to be the best predictor of hepatic decompensation (HD).^2^^,^^3^ Although the hepatic venous pressure gradient (HVPG) remains the gold standard for assessing CSPH, this procedure is invasive, not routinely performed, and is affected by a considerable operator-dependent accuracy.^4^

To face this, alternative non-invasive tools (NITs) have been progressively developed, including Fibrosis-4 (FIB-4),^5^ Albumin-Bilirubin score (ALBI),^6^ ALBI-FIB-4,^7^ the ANTICIPATE,^4^ (subsequently adapted for obese patients with non-alcoholic steatohepatitis [NASH] by incorporating body mass index [BMI] [ANTICIPATE ± NASH]),^8^ the von Willebrand factor (VWF) antigen-to-platelet (PLT) count ratio (VITRO),^9^ liver stiffness measurement (LSM)-to-platelet ratio (LSM/PLTr),^10^ and the red cell distribution width-to-platelet ratio (RPR).^11^ Recently, *Reiberger et al.* revealed NITs provide prognostic information comparable to the HVPG to predict decompensation.^12^

Noteworthily, except for ALBI, the PLT count represents the common denominator and a cornerstone variable of all these models.^4^^,^^5^^,^^7^, ^8^, ^9^, ^10^, ^11^ The physio-pathological rationale is the recurrence of thrombocytopenia as a frequent hematological disorder in advanced chronic liver disease (ACLD) patients, determined by multifactorial elements (*e.g.,* systemic inflammation and reduced poiesis), with a predominant role of hypersplenism, mainly due to portal hypertension–related splenomegaly.^13^^,^^14^ The counterevidence is that in splenectomized individuals, the absence of splenic pooling can result in a normal PLT count compared to the standard cut-offs.^15^

Nevertheless, in ACLD, the prevalence of splenomegaly is about 60–65%,^16^ and its absence cannot be used to rule out portal hypertension effectively.^17^ Indeed, in ACLD, the prevalence of thrombocytopenia has been reported at approximately 78%^13^ and hypersplenism occurs in up to 61% of cases.^18^ These data highlight a non-negligible proportion of ACLD patients with CSPH not presenting with splenomegaly and, consequently, may not develop hypersplenism-related thrombocytopenia.

Alarmingly, this evidence potentially questions the reliability of the most used NITs suggesting these could exclude a significant part of patients from screening and prophylaxis programs, potentially leading to fatal decompensations, designing a scenario where the evaluation of CSPH is based on PLT count without considering spleen size.

Metabolic dysfunction–associated steatotic liver disease (MASLD), with its clinical and pathophysiological implications with obesity and other comorbidities of metabolic syndrome, currently represents a predominant cause of ACLD worldwide with a severe socioeconomic burden.^19^^,^^20^ Interestingly, previous research has suggested a close relationship between spleen size, fat-free mass, and obesity.^21^ More relevantly, MASLD-related cACLD may progress more rapidly than other etiologies and present a relatively earlier HD.^22^^,^^23^

Considering this background, evaluating the relative performance of currently available NITs in predicting HD in MASLD-related cACLD patients by stratifying for spleen size represented the main scope of the present research.

## PATIENTS AND METHODS

### Experimental Design

In this observational study, MASLD-related cACLD-affected outpatients, naïve for the administration of non-selective beta-blockers (NSBBs) and the onset of previous LRDEs, receiving a screening esophagogastroduodenoscopy showing esophageal varices were enrolled and considered CSPH affected.^24^ In parallel, a sub-cohort of asplenic (ASP) individuals (patients with a medical history of splenectomy or with splenic agenesis) presenting the same clinical specifications (*i.e.*, MASLD-related cACLD and CSPH manifest for esophageal varices) was also recruited.

According to the Baveno consensus, ACLD was defined by LSM values >15 kPa.^25^ Subsequently, non-ASP individuals received an ultrasonographic (US) assessment of the spleen size, evaluating the diameter and the organ area.

**Figure 1:**
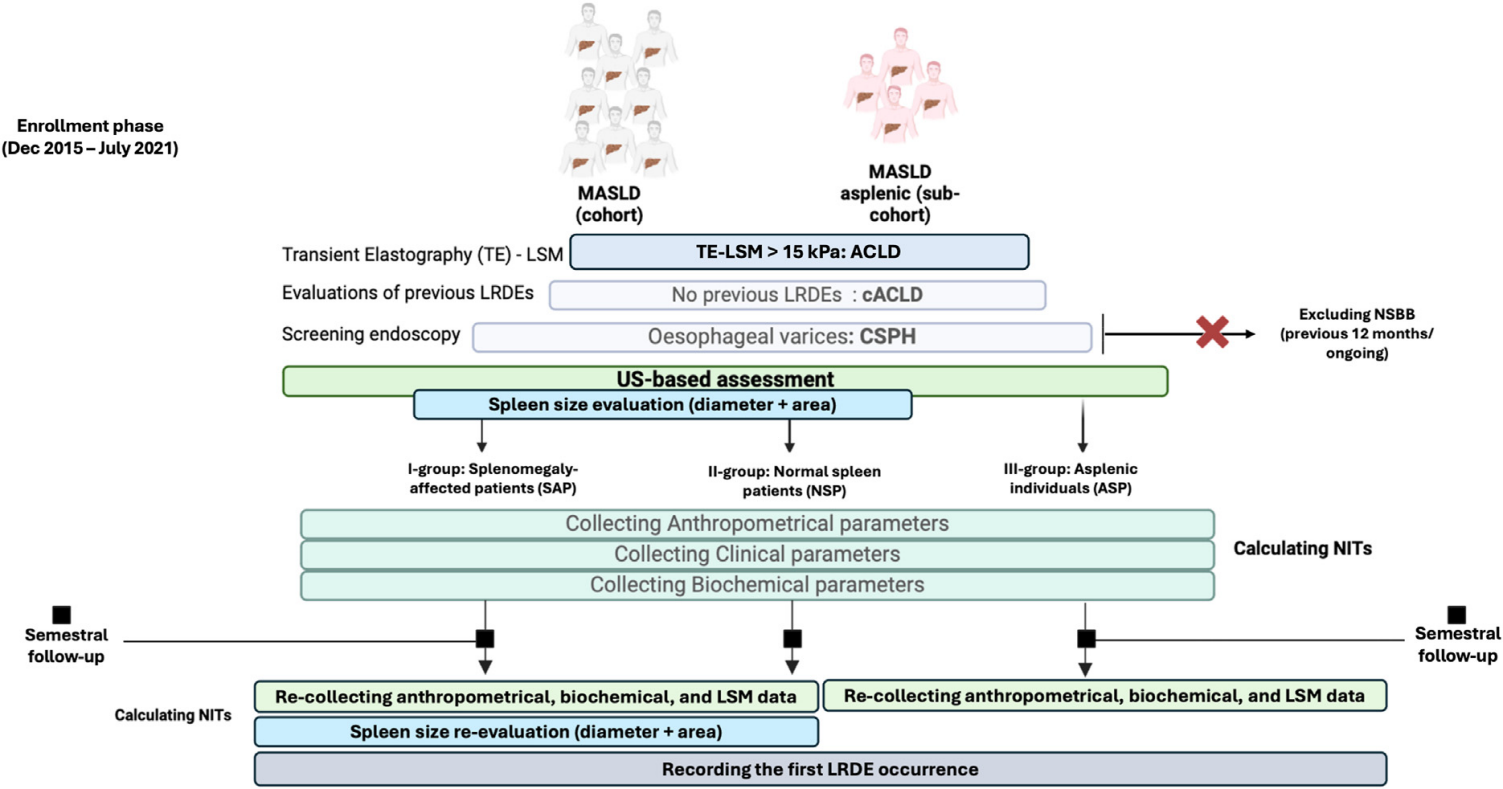
Experimental design flowchart. Non-invasive tools (NITs) included ALBI, FIB-4, ALBI-FIB-4, LSM/PLTr, RPR, ANTICIPATE ± NASH. LRDEs, liver-related decompensation events; NSBB, non-selective beta-blocker; CSPH, clinically significant portal hypertension; cACLD, compensated advanced chronic liver disease; LSM, liver stiffness measurement; TE-LSM: Transient Elastograpgy liver stiffness measurement; NASH, non-alcoholic steatohepatitis; LSM/PLTr, liver stiffness measurement-to-platelet ratio; FIB-4, Fibrosis-4; ALBI, Albumin-Bilirubin score; MASLD, metabolic dysfunction–associated steatotic liver disease; RPR, red cell distribution width-to-platelet ratio.

Considering the European Federation of Societies for Ultrasound in Medicine and Biology (EFSUMB) recommendations,^26^ a spleen diameter >12 cm, simultaneously with a spleen area ≥45 cm^2^,^26^, ^27^, ^28^ was adopted to define splenomegaly. Finally, three study groups were identified: I: splenomegaly-affected patients (“**SAPs**”), II: normal spleen patients (“**NSPs**”), and III: ASP individuals (“**ASP**”). At the enrollment, clinical, anthropometric, and biochemical data were collected. According to the available clinical practice guidelines on primary prophylaxis in CSPH, the administration of NSBBs (propranolol or carvedilol) at a low dosage, subsequently opportunely titrated to achieve the tailored-target dosage, was started (*see the next dedicated section for details*).^25^ The entire population was followed up semiannually for 3 years. During this period, anthropometrical, biochemical, and LSM data were recollected, spleen size was US-reassessed (in groups I and II), the NSBB effectiveness was evaluated (*see the next dedicated section for details*), and any eventual LRDE (ascites, variceal bleeding, hepatic encephalopathy [at least mild, i.e., ≥grade 1 according to West Haven classification],^29^ jaundice, and acute bacterial infections requiring hospitalization)^1^ with the relative time of occurrence was recorded. Notably, for each patient, the first LRDE occurrence represented the end of follow-up. The anthropometric and biochemical variables were used *a posteriori* to compute (relative to baseline and follow-up evaluations) the ALBI score^30^ and calculate the following PLT count–based NITs: FIB-4,^5^ ALBI-FIB-4,^7^ LSM/PLTr,^10^ RPR,^11^ and the estimation of individual CSPH risk according to the ANTICIPATE, or in obese patients, the ANTICIPATE ± NASH model.^8^^,^^10^ Figure 1 reports the experimental flowchart (Figure 1).

In MASLD-related cACLD individuals presenting CSPH, the correlation between spleen size and PLT count was evaluated as the propaedeutic study outcome. In this setting of patients, the comparison of the performance of ALBI and PLT count–based NITs in predicting the first LRDE occurrence (*i.e.*, HD) at 3 years in the three study groups (“SAP,” “NSP,” and “ASP”) constituted the primary outcome of the present research.

The “STrengthening the Reporting of Observational Studies in Epidemiology” checklist is reported in Supplementary File 1 (Supplementary File 1).

### Patients

This prospective longitudinal study complies with the ethical guidelines of the Declaration of Helsinki (1975) and was approved by the ethical committee of the University of Campania “L. Vanvitelli” in Naples (prot n. 0018123/2015). The enrollment was carried out at the Hepato-Gastroenterology Division of the University of Campania “Luigi Vanvitelli” between December 2015 and July 2021. Inclusion criteria were (a) age between 18 and 70 years; (b) ACLD related to a proven clinical history of non-alcoholic fatty liver disease (NAFLD), respecting the relatively updated multi-society Delphi consensus proposed criteria for MASLD^20^; (c) asplenia secondary to splenectomy or splenic agenesis (exclusively for the study sub-cohort); (d) CSPH manifest for endoscopic evidence of variceal varices^24^; and (e) availability to sign informed consent. Transient elastography was adopted to determine LSM, and, according to the Baveno VI consensus, LSM values >15 kPa identified ACLD.^31^ Regarding the diagnosis of MASLD, the recently proposed nomenclature update with the relative diagnostic criteria (from NAFLD, to metabolic dysfunction–associated fatty liver disease [MAFLD], and lastly to MASLD^20^^,^^32^^,^^33^) was considered. All the enrolled NAFLD individuals presented the updated diagnostic criteria and were ultimately considered MASLD patients.^20^

Exclusion criteria were (a) chronic liver disorders otherwise than MASLD; (b) previous LRDEs in the last 12 months without obtained recompensation^31^^,^^34^; (c) other causes determining CSPH (*i.e.,* splenic/portal vein thrombosis, schistosomiasis, Budd-Chiari syndrome, and arteriovenous fistula); (d) administration of NSBBs (including carvedilol, propranolol, and nadolol), in the previous 12 months and/or ongoing; (e) collagenopathies, infections, hemolytic anemia, storage diseases, and any other pathology that could lead to splenomegaly from a different cause (exclusively for the study cohort); (f) cancer/leukemia/lymphoma diagnosis; (g) pregnancy; (h) acute or chronic kidney diseases with a glomerular filtration rate <30 mL/min; and (i) psychological/psychiatric problems that could have invalidated the informed consent.

After signing the informed consent, we consecutively enrolled MASLD-cACLD patients presenting CSPH, as well as a sub-cohort of ASP MASLD-cACLD with CSPH individuals. A US-assessed spleen diameter >12 cm, simultaneously with a spleen area ≥45 cm^2^, according to EFSUMB recommendations,^26^, ^27^, ^28^ defined splenomegaly. Ultimately, three groups were identified: I: “**SAP**”, II: “**NSP**”, and ASIII: “**ASP”** (Figure 1).

At the baseline, we investigated both the immediate and the remote pathological history of each patient. Anthropometrical evaluations were also performed, including the assessment of systolic blood pressure and diastolic blood pressure (mmHg), as well as BMI calculation by dividing the weight (kg) by the square of height (m) and body surface area (BSA), calculated using Mosteller formula: BSA = √[(height in cm × weight in kg)/(3600)].

The collected biochemical variables included PLT count (u/microL), red blood cell distribution width – standard deviation (RDW-SD) (fL), aspartate aminotransferase (U/l), alanine aminotransferase (U/l), total bilirubin (mg/dL), fasting plasma glucose (mg/dl), glycosylated hemoglobin (%), total cholesterol (mg/dl), high-density lipoprotein cholesterol, low-density lipoprotein cholesterol, triglycerides (mg/dl), serum albumin (g/dl), international normalized ratio, C-reactive protein (CRP) (mg/dl), and creatinine (mg/dl). For each patient, the Child-Pugh (CP) score and the Model for End-Stage Liver Disease (MELD) score were determined,^35^ as well as the anthropometrical and biochemical data were adopted *a posteriori* to calculate the following NITs: ALBI,^30^ FIB-4,^5^ ALBI-FIB-4,^7^ LSM/PLTr,^10^ RPR,^11^ and the estimation of individual CSPH risk according to the ANTICIPATE, or in obese patients, the ANTICIPATE-NASH (ANTICIPATE ± NASH).^8^ Lastly, patients were followed up every six months during the study period to recollect anthropometrical, biochemical, and LSM data, reassess spleen size (in I and II), evaluate the NSBB effectiveness (*see in the following paragraphs*), and record eventual occurred LRDEs. LSM, spleen size, and NIT determination methods are detailed in Supplementary File 2 and Supplementary Figure 1.

### NSBB Administration and Evaluation of Effectiveness

All individuals with endoscopic evidence of CSPH (*i.e*., small and medium-large esophageal varices) started an NSBB-based primary prophylaxis regimen (propranolol or carvedilol).^25^^,^^36^ For each patient, NSBB dosage was progressively incremented, until reaching and observing a reduction of 25% of the baseline heart rate (HR) (beats per minute - bpm) or HR value = 55 bpm within the subsequent follow-up visit (achievement of “tailored-target dose”).^25^^,^^36^

Despite it constitutes a surrogate not entirely faithfully expressing the effective response to NSBB, in consideration of HPVG (gold standard) unavailability, the achievement of a “tailored-target dose” was adopted to discriminate patients “NSBB-protected” (“effectiveness”) from individuals “NSBB-not protected” (“not-effectiveness”).

### Statistical Analysis

The Kolmogorov-Smirnov test for normality was performed to evaluate if the parametric or non-parametric analysis should be applied. Continuous data were described as mean and standard deviations, while categorical variables were described as n (%). Mann-Whitney and t-test for independent groups, the Kruskal-Wallis test, or analysis of variance test with *post hoc* Tukey analysis, according to the non-normal or normal distribution, were performed to compare the continuous variables.

The chi-squared test (or Fisher's exact test) was performed to compare the frequency distribution relative to the categorical variables among the three study groups. Linear regression analysis was adopted to evaluate the relationship (R) between continuous variables.

The log-rank test analysis, with Kaplan-Meier curve comparison, was adopted to determine the risk (hazard ratio) (HR) and compare the cumulative incidence proportion and incidence ratio rate of LRDEs at 1, 2, and 3 years. Univariate and multivariate competing risk regression analyses for HD during follow-up (adjusted for sex, age, BMI, diabetes, albumin levels, MELD, and NSBB effectiveness) defined the variables independently predicting adjusted sub-distribution hazard ratio (aSHR) LRDEs.

Receiving operator curve (ROC) with the identification of the best cut-off (BCO) through the Youden index and time-dependent ROC analysis were adopted to determine the performance of ALBI and other PLT count–based NITs in the prediction of a 3-year first LRDE. The relative areas under the curve (AUCs), as well as AUC under the time-dependent ROC, were calculated. Time-dependent AUCs were compared at 12 months, 18 months, and 24 months of follow-up by using the DeLong test.

The sample size was estimated by using a chi-squared test confronting two independent proportions, singularly predicting a 30% difference in the incidence of 3-years HD in patients presenting an ALBI score over the ROC-identified BCO in group II compared with group I, as well as in group III compared with group I (significance: 0.05, type II error: 0.1; power: 0.9) (Stata14 for macOS, manufactured by StataCorp LLC) and resulted in n 50 individuals for groups I and II and 25 for group III.

Statistical significance was defined as a *P* value <0.05 in a two-tailed test with a 95% confidence interval. GraphPad Prism vs.10.1.1 was used to perform the analysis.

## RESULTS

### Baseline Patient Characteristics

A total of 148 patients and 27 ASP (2 with agenesis and 25 splenectomized after traumas) (III: ASP) individuals with MASLD-related cACLD and CSPH were enrolled. Of the 148 patients, 91 (61.48%) presented splenomegaly (I-SAP, Spleen Area 64.56 ± 7.32 cm^2^), whereas 57 (38.52%) had a normal spleen size (II: NSP, spleen area: 39.92 ± 3.48 cm^2^). Patient characteristics are reported in Table 1.

**Table 1:** Baseline Characteristics of the Study Groups.

Demographic, anthropometric, and clinical data				
	SAPs (n: 91) (I)	NSPs (n: 57) (II)	ASP patients (n: 27) (III)	*P* value
Gender M/F (number and %)	52 (57.14%) 39 (42.86%)	34 (59.64%) 23 (40.36%)	15 (55.56%) 12 (44.44%)	I vs II^a^: n.s. II vs III^a^: n.s. I vs III^a^: n.s.
Age (mean ± SD)	61.37 ± 10.9	61.98 ± 14.2	64.74 ± 13.2	I vs II^a^: n.s. II vs III^a^: n.s. I vs III^a^: n.s.
Height (cm) (mean ± SD)	169.8 ± 5.79	170.6 ± 5.51	171.3 ± 5.75	I vs II^a^: n.s. II vs III^a^: n.s. I vs III^a^: n.s.
Weight (Kg) (mean ± SD)	84.62 ± 3.59	84.65 ± 3.13	84.26 ± 2.96	I vs II^a^: n.s. II vs III^a^: n.s. I vs III^a^: n.s.
BMI (mean ± SD)	30.78 ± 3.00	30.60 ± 3.81	30.84 ± 3.05	I vs II^a^: n.s. II vs III^a^: n.s. I vs III^a^: n.s.
BSA (mean ± SD)	1.99 ± 0.05	2.00 ± 0.04	2.00 ± 0.05	I vs II^a^: n.s. II vs III^a^: n.s. I vs III^a^: n.s.
Child-Pugh—A (number and %)	59 (64.83%)	36 (63.15%)	22 (81.48%)	I vs II^a^: n.s. II vs III^a^: n.s. I vs III^a^: n.s.
Child-Pugh—B (number and %)	32 (35.16%)	21 (36.84%)	5 (18.51%)	I vs II^a^: n.s. II vs III^a^: n.s. I vs III^a^: n.s.
Obesity (number and %)	78 (85.71%)	49 (85.96%)	23 (85.18%)	I vs II^a^: n.s. II vs III^a^: n.s. I vs III^a^: n.s.
T2DM (number and %)	53 (58.24%)	31 (54.38%)	16 (59.26%)	I vs II^a^: n.s. II vs III^a^: n.s. I vs III^a^: n.s.
Dyslipidemia (number and%)	48 (52.74%)	28 (49.12%)	13 (48.14%)	I vs II^a^: n.s. II vs III^a^: n.s. I vs III^a^: n.s.
Hypertension (number and %)	49 (53.84%)	30 (52.63%)	15 (55.55%)	I vs II^a^: n.s. II vs III^a^: n.s. I vs III^a^: n.s.
Systolic blood pressure	133.1 ± 12.17	131.0 ± 12.24	130.6 ± 9.39	I vs II^a^: n.s. II vs III^a^: n.s. I vs III^a^: n.s.
Diastolic blood pressure (mean ± SD)	88.79 ± 7.35	88.26 ± 9.97	87.67 ± 8.77	I vs II^a^: n.s. II vs III^a^: n.s. I vs III^a^: n.s.
Spleen area (cm^2^) (mean ± SD)	64.56 ± 7.326	39.92 ± 3.488	N.A.	I vs II^b^: **<0.0001** II vs III^b^:/ I vs III^b^:/
Spleen diamet. (cm) (mean ± SD)	18.69 ± 1.39	10.63 ± 0.86	N.A.	I vs II^b^: **<0.0001** II vs III^b^:/ I vs III^b^:/

Biochemical parameters				
Variables (mean ± SD)	SAPs (n: 91) (I)	NSPs (n: 57) (II)	ASP patients (n: 27) (III)	*P* value
AST (u/l)	49.58 ± 54.2	49.04 ± 54.5	36.74 ± 19.8	I vs II^a^: n.s. II vs III^a^: n.s. I vs III^a^: n.s.
ALT (u/l)	72.95 ± 146	66.04 ± 161	38.19 ± 20.9	I vs II^a^: n.s. II vs III^a^: n.s. I vs III^a^: n.s.
Bilirubin (mg/dl)	2.05 ± 1.93	1.57 ± 1.10	1.69 ± 0.98	I vs II^a^: n.s. II vs III^a^: n.s. I vs III^a^: n.s.
PLT count (u/microL)	109.2 ± 26.7	169 ± 30.7	176.7 ± 14.6	I vs II^b^: **<0.0001** II vs III^b^: n.s. I vs III^b^:**<0.0001**
RDW (SD)	53.84 ± 10.2	60.03 ± 10.3	68.51 ± 7.22	**I vs II**^b^**: 0.0001** **II vs III**^b^**: 0.0002** **I vs III**^b^**: <0.0001**
Albumin (g/dl)	3.56 ± 0.65	3.66 ± 0.80	3.54 ± 0.50	I vs II^a^: n.s. II vs III^a^: n.s. I vs III^a^: n.s.
INR	1.29 ± 0.37	1.23 ± 0.31	1.25 ± 0.33	I vs II^a^: n.s. II vs III^a^: n.s. I vs III^a^: n.s.
Creatinine (mg/dl)	1.78 ± 4.95	1.02 ± 0.59	1.01 ± 0.22	I vs II^a^: n.s. II vs III^a^: n.s. I vs III^a^: n.s.
CRP (mg/dl)	1.88 ± 0.41	1.80 ± 0.46	1.99 ± 0.33	I vs II^a^: n.s. II vs III^a^: n.s. I vs III^a^: n.s.
FPG (mg/dl)	122 ± 18.37	119 ± 15.93	119 ± 16.89	I vs II^a^: n.s. II vs III^a^: n.s. I vs III^a^: n.s.
Cholesterol (mg/dl)	190 ± 41.52	180 ± 48.38	188 ± 58.21	I vs II^a^: n.s. II vs III^a^: n.s. I vs III^a^: n.s.
HDL (mg/dl)	41.7 ± 10.23	43.5 ± 9.79	42.5 ± 9.96	I vs II^a^: n.s. II vs III^a^: n.s. I vs III^a^: n.s.
LDL (mg/dl)	124.9 ± 40.52	126.2 ± 36.95	125.1 ± 40.93	I vs II^a^: n.s. II vs III^a^: n.s. I vs III^a^: n.s.
TG (mg/dl)	155 ± 69.98	144 ± 51.17	152 ± 68.11	I vs II^a^: n.s. II vs III^a^: n.s. I vs III^a^: n.s.
HbA1c (%)	6.44 ± 0.40	6.42 ± 0.45	6.39 ± 0.36	I vs II^a^: n.s. II vs III^a^: n.s. I vs III^a^: n.s.

Non-invasive tools for liver disease severity assessment				
Variables (mean ± SD)	SAPs (n: 91) (I)	NSPs (n: 57) (II)	ASP patients (n: 27) (III)	*P* value
LSM (kPa)	23.5 ± 3.26	23.3 ± 3.65	23.7 ± 2.20	I vs II^a^: n.s. II vs III^a^: n.s. I vs III^a^: n.s.
Child-Pugh (point)	6.19 ± 1.18	6.22 ± 1.23	5.92 ± 0.87	I vs II^a^: n.s. II vs III^a^: n.s. I vs III^a^: n.s.
MELD (point)	7.75 ± 0.74	7.49 ± 0.42	7.35 ± 0.76	I vs II^a^: n.s. II vs III^a^: n.s. I vs III^a^: n.s.
LSM/PLT ratio	0.236 ± 0.10	0.141 ± 0.31	0.135 ± 0.01	I vs II^b^: **<0.0001** II vs III^b^: n.s. I vs III^b^: **<0.0001**
RPR (point)	0.538 ± 0.23	0.372 ± 0.11	0.389 ± 0.04	I vs II^b^: **<0.0001** II vs III^b^: n.s. I vs III^b^: **0.0002**
ANTICIPATE ± NASH (logit)	−3.45 ± 0.45	−4.32 ± 0.46	−4.34 ± 0.24	I vs II^b^: **<0.0001** II vs III^b^: n.s. I vs III^b^: **<0.0001**
FIB-4 (point)	2.86 ± 1.50	2.77 ± 2.20	2.70 ± 1.76	I vs II^b^: **0.0148** II vs III^b^: n.s. I vs III^b^: **0.0121**
ALBI (point)	−2.44 ± 0.63	−2.38 ± 0.67	−2.47 ± 0.47	I vs II^a^: n.s. II vs III^a^: n.s. I vs III^a^: n.s.

Statistically significant differences (*P* < 0.05) are reported in bold.

n.s., not statistically significant; SAP, splenomegaly-affected patient; NSP, normal-spleen patient; ASP, asplenic; BMI, body mass index; BSA, body surface area; T2DM, type 2 diabetes mellitus; AST, aspartate aminotransferase; ALT, alanine aminotransferase; PLT, platelet; INR, international normalized ratio; CRP, C-reactive protein; FPG, fasting plasma glucose; HbA1c, glycosylated hemoglobin; HDL, high-density lipoprotein cholesterol; LDL, low-density lipoprotein cholesterol; TG, triglycerides; RDW-SD, red cell distribution width—standard deviation; RPR, red cell distribution width-to-platelet ratio; LSM, liver stiffness measurement; FIB-4, Fibrosis-4; ALBI, Albumin-Bilirubin score; MELD, Model for End-Stage Liver Disease; NASH, non-alcoholic steatohepatitis.

a Chi-squared test analysis.

b Mann-Whitney U test.

By comparing for sex, the spleen area measurements were similar both in patients with and without splenomegaly (SAP: male 65.39 ± 7.40, female 63.46 ± 7.16, *P*: 0.1955; NSP: male 40.12 ± 3.45, female 39.63 ± 3.59, *P*: 0.7255), as well as the overall prevalence of splenomegaly was not significantly different according to gender (chi-square, *P*: 0.3043).

NSPs and ASP individuals presented similar features, while substantial differences were highlighted by comparing SAPs with NSPs, as well as SAPs with ASP patients, in the following parameters: PLT count (SAPs vs NSPs and SAPs vs ASP patients, both *P* < 0.0001), FIB-4 (SAPs vs NSPs, *P*: 0.0149; SAPs vs ASP patients, *P*: 0.0121), LSM/PLTr (SAPs vs NSPs and SAP vs ASP patients, both *P* < 0.0001), RDW-SD (SAPs vs NSPs, *P*: 0.0001; SAPs vs ASPs, *P* < 0.0001), RPR (SAPs vs NSPs, *P* < 0.0001; SAPs vs ASP patients, *P*: 0.0002), and ANTICIPATE ± NASH (SAPs vs NSPs and SAPs vs ASP patients, both *P* < 0.0001). Regarding hepatofunctionality, liver disease progression status, and systemic inflammation, no statistically significant differences emerged in CP, MELD, LSM, and CRP among the groups (Table 1).

Concerning CSPH, based on endoscopically evidenced varices features, no statistically significant differences were reported in terms of the frequency distribution of varices dimension by comparing the study groups (SAP: small varices: 67 [73.62%], medium-large varices: 24 [26.38%]; NSP: small varices: 41 [71.92%], medium-large varices: 16 [28.08%]; ASP: small varices: 19 [70.37%], medium-large varices: 8 [29.63%], chi-square *P* > 0.05 for all).

### The Spleen Size—Platelet Count Relationship in Patients With and Without Splenomegaly

**Figure 2:**
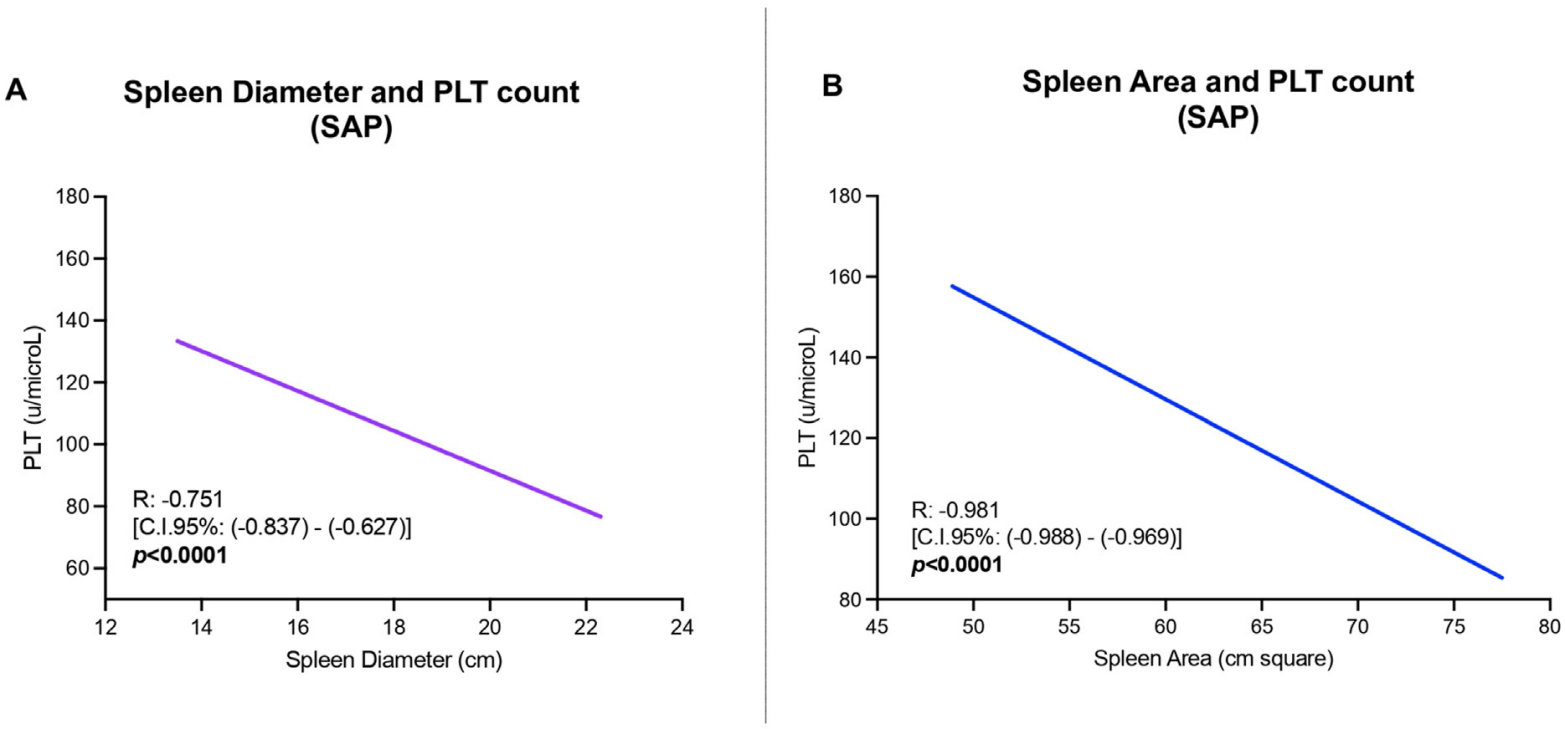
Spleen diameter (cm)—PLT count (u/microL) (**A**) and spleen area (cm^2^)—PLT count (u/microL) (**B**) relationships in MASLD-cACLD SAP patients (**A**). Linear regression analysis. CSPH, clinically significant portal hypertension; cACLD, compensated advanced chronic liver disease; PLT, platelet. SAP, splenomegaly-affected patient; MASLD, metabolic dysfunction–associated steatotic liver disease; cACLD, compensated advanced chronic liver disease; CI, confidence nterval; R, relationship.

Considering the baseline spleen diameter (cm), a significantly inverse correlation with baseline PLT count was reported exclusively in patients presenting splenomegaly (SAP: spleen diameter – PLT, R: −0.751, *P* < 0.0001; NSP: spleen diameter – PLT, R: 0.062, *P*: 0.646) (Figure 2, panel A).

Moreover, in these individuals, in contrast with individuals not showing splenomegaly (NSP: R: 0.004, *P*: 0.978), an inverse significant punctual relationship between the spleen area (cm^2^) and the PLT count (u/microL) was also observed (SAP: R: −0.981; *P* < 0.0001) (Figure 2, Panel B).

### Follow-up: Spleen Size Modifications, NSBB Effectiveness, and Decompensation Occurrence

During the observation period, 16 (9.14%) patients (8 [8.79%] SAP, 5 [8.77%] NSP, and 3 [11.11%] ASP) were lost and did not complete follow-ups.

Spleen size remained constant in SAPs and NSPs throughout the study, and none of the NSPs developed splenomegaly: the six-month trend of splenic diameter and area is observable in Supplementary Figure 2. Globally, the NSBB effectiveness was reported in 151 patients (SAP: 78; NSP: 49; ASP:24) (effectiveness rate: 86.28%). The evaluation of NSBB effectiveness according to the administration of propranolol or carvedilol is detailed in Supplementary Figure 3.

**Figure 3:**
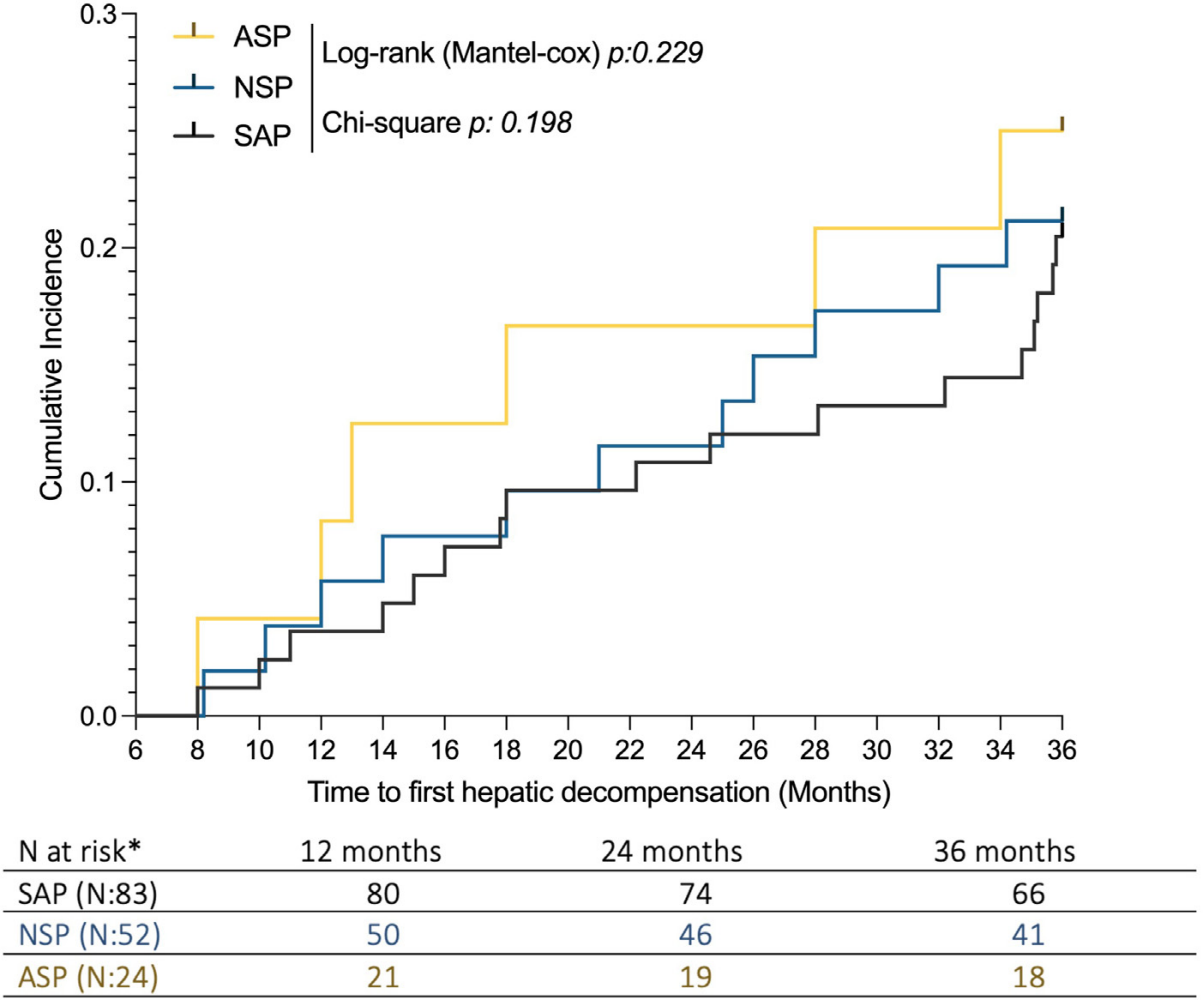
Cumulative incidence of first hepatic decompensation across the study population groups. MASLD-cACLD-CSPH SAP (black), NSP (blue), and ASP individuals (yellow). Log-rank with chi-square test analysis; statistically significant differences (*P* < 0.05). CSPH, Clinically significant portal hypertension; cACLD, compensated advanced chronic liver disease; SAP, splenomegaly-affected patient; NSP, normal-spleen patient; ASP, asplenic; MASLD, metabolic dysfunction–associated steatotic liver disease. ∗16 patients did not complete follow-ups.

During a median follow-up of 35.8 (8–36) months, the first HD was observed in 17 (20.48%) SAPs (median time of decompensation: 22.20 months), 11 (21.15%) NSPs (21 months), and 6 (25%) ASP individuals (16.50 months) (log-rank, *P*: 0.229). In SAPs, the cumulative incidence of HD at 1 and 2 years was 3.29% and 9.89%, respectively, whereas in NSPs, it was 3.51% and 10.52% (chi-square, *P*: 0.198) (Figure 3). The distribution of the LRDEs among the groups is detailed in Supplementary Table 1.

### Variables Predicting Decompensation in the Three Study Groups

In MASLD-cACLD SAP presenting CSPH, albumin (*P* < 0.0001), bilirubin (*P*: 0.001), BMI (*P*: 0.04), MELD (*P*: 0.001), PLT count (*P*: 0.03), spleen area (*P*: 0.02), LSM (*P*: 0.03), ALBI (*P* < 0.0001), and all the PLT count–based NITs (FIB-4, ALBI-FIB-4, RPR, LSM/PLT ratio, and ANTICIPATE ± NASH) (all *P* < 0.0001) were evidenced as variables predicting the decompensation, in contrast with the presence of diabetes mellitus (*P*: 0.612). The multivariate analysis revealed the ALBI (*P*: 0.003) and the PLT count–based NITs (FIB-4 *P*: 0.002], ALBI-FIB-4 [*P* < 0.0001], RPR [*P* < 0.0001], LSM/PLTr [*P*: 0.002], and ANTICIPATE ± NASH [*P* < 0.001]) as the exclusive variables significantly predicting the HD in these individuals, independently from sex, age, BMI, albumin levels, diabetes mellitus, MELD, and NSBB effectiveness. Contrarily, the spleen area was not significantly associated with this outcome (Table 2).

**Table 2:** Univariate and Multivariate Analyses for Decompensation in Splenomegaly-Affected, Normal-Spleen, and Asplenic Patients.

Variable	Splenomegaly-affected patients				Normal-spleen patients			
	Univariate^a^		Multivariate^b^		Univariate^a^		Multivariate^b^	
	SHR (95% CI 95%)	*P* value	aSHR (95% CI)	*P* value	SHR (95% CI)	*P* value	aSHR (95% CI)	*P* value
Albumin (g/dl)	0.673 (0.258–0.857)	<0.0001	–	–	0.721 (0.431–0.841)	<0.0001	–	–
Bilirubin (mg/dl)	1.131 (1.078–1.450)	0.001	–	–	1.112 (1.098–1.250)	0.001	–	–
MELD (point)	1.112 (1.091–1.782)	0.001	–	–	1.140 (1.073–1.881)	0.002	–	–
PLT (10^3^/mm^3^)	0.880 (0.781–0.932)	0.03	–	–	0.760 (0.631–0.834)	0.01	–	–
BMI (kg/m^2^)	1.091 (0.874–1.345)	0.04	–	–	1.380 (1.061–1.495)	0.04	–	–
T2DM (yes vs no)	1.311 (0.932–2.185)	0.612	–	–	1.270 (0.951–1.935)	0.523	–	–
LSM (kPa)	1.872 (1.581–2.025)	0.03	–	–	1.720 (1.592–2.131)	0.02	–	–
Spleen area (cm^2^)	1.073 (0.783–1.162)	0.02	–	–	0.890 (0.798–1.026)	0.001	0.870 (0.833–1.108)	**<0.0001**
ALBI (point)	1.213 (1.027–1.384)	<0.0001	1.188 (1.091–1.216)	**0.003**	1.323 (1.137–1.402)	<0.0001	1.181 (1.082–1.193)	**0.002**
FIB-4 (point)	1.207 (1.148–1.341)	<0.0001	1.113 (1.088–1.171)	**0.002**	1.188 (1.167–1.921)	<0.0001	1.102 (1.092–1.165)	**0.03**
ALBI-FIB-4 (point)	1.329 (1.276–1.459)	<0.0001	1.221 (1.199–1.252)	**<0.0001**	1.285 (1.210–1.319)	<0.0001	1.164 (1.141–1.241)	**0.001**
RPR (unit)	1.340 (1.184–1.726)	<0.0001	1.192 (1.018–1.292)	**<0.0001**	1.271 (1.085–1.956)	<0.0001	1.172 (0.881–1.122)	**0.002**
LSM/PLT ratio (unit)	1.257 (0.992–1.458)	<0.0001	1.143 (0.985–1.351)	**0.002**	1.135 (0.909–1.286)	<0.0001	1.047 (0.995–1.101)	**0.01**
ANTICIPATE ± NASH(c)	2.974 (1.936–3.241)	<0.0001	2.851 (1.790–3.005)	**<0.001**	2.013 (1.896–3.312)	<0.0001	1.224 (1.390–2.225)	**0.001**

Variable	Asplenic (ASP) patients			
	Univariate^a^		Multivariate^b^	
	SHR (95% CI)	*P* value	SHR (95% CI)	*P* value
Albumin (g/dl)	0.745 (0.562–0.827)	<0.0001	–	–
Bilirubin (mg/dl)	1.127 (1.10–1.216)	0.001	–	–
MELD (point)	1.132 (1.093–1.202)	0.003	–	–
PLT (10^3^/mm^3^)	0.753 (0.672–0.871)	0.001	–	–
BMI (kg/m^2^)	1.234 (1.093–1.388)	0.03	–	–
T2DM (yes vs no)	1.194 (0.831–1.755)	0.413	–	–
LSM (kPa)	1.534 (1.472–2.104)	0.03	–	–
Spleen area (cm^2^)	–	–	–	–
ALBI (point)	1.221 (1.134–1.512)	<0.0001	1.273 (1.199–1.305)	**0.002**
FIB-4 (point)	1.159 (1.237–1.938)	<0.0001	1.122 (1.102–1.139)	n.s.
ALBI-FIB-4 (point)	1.261 (1.205–1.324)	<0.0001	1.094 (1.073–1.117)	**0.041**
RPR (unit)	1.248 (1.057–1.839)	<0.0001	1.003 (0.871–1.129)	n.s.
LSM-to-PLT ratio (unit)	1.132 (0.985–1.184)	<0.0001	1.025 (0.991–1.121)	n.s.
ANTICIPATE ± NASH(c)	2.091 (1.992–2.517)	<0.0001	2.023 (1.782–2.124)	n.s.

Statistically significant differences (*P* < 0.05) (in bold in the multivariate model).

ASP, asplenic; CSPH, clinically significant portal hypertension; cACLD, compensated advanced chronic liver disease; aSHR, adjusted sub-distribution hazard ratio; CI, confidence interval; T2DM, type 2 diabetes mellitus; PLT, platelet; RDW, red cell distribution width; RPR, red cell distribution width-to-platelet ratio; LSM, liver stiffness measurement; n.s., not statistically significant; NASH, non-alcoholic steatohepatitis; MELD, Model for End-Stage Liver Disease; NBBB, non-selective beta-blocker; FIB-4, Fibrosis-4; ALBI, Albumin-Bilirubin score; BMI: body mass index.

a Univariate competing risk regression model (adjusted for sex, age, BMI, diabetes, albumin levels, MELD, and NSBB effectiveness).

b Multivariate competing risk models (adjusted for sex, age, BMI, diabetes, albumin levels, MELD, and NSBB effectiveness).

c CSPH probability.

In MASLD-cACLD NSPs presenting CSPH, albumin (*P* < 0.0001), bilirubin (*P*: 0.001), BMI (*P*: 0.04), MELD (*P*: 0.002), PLT count (*P*: 0.01), LSM (*P*: 0.02), ALBI (*P* < 0.0001), and all the PLT count–based NITs (all *P* < 0.0001) were variables predicting the decompensation.

The spleen area and diabetes mellitus were, respectively, inversely associated (sub-distribution hazard ratio: 0.890; *P*: 0.001) and not associated (*P*: 0.523) with this outcome.

The multivariate analysis confirmed the ALBI (*P*: 0.002) and PLT count–based NITs (FIB-4 [*P*: 0.03], ALBI-FIB-4 [*P*: 0.001], RPR[*P*: 0.002], LSM/PLT ratio [*P*: 0.01], and ANTICIPATE ± NASH [*P*: 0.001]) as significantly predicting the HD in these individuals, highlighting, by comparing to patients with splenomegaly, a reduced influence (*i.e.,* a relatively lower aSHR) of FIB-4, ALBI-FIB-4, RPR, LSM/PLTr, and ANTICIPATE ± NASH on the outcome. Moreover, in contrast with the findings reported in patients with splenomegaly, the spleen area was confirmed as inversely associated (aSHR: 0.870), also emerging as the variable significantly (*P* < 0.0001) predicting the decompensation (Table 2).

In ASP individuals, similarly to NSP, albumin (*P* < 0.0001), bilirubin (*P*: 0.001), BMI (*P*: 0.03), MELD (*P*: 0.003), PLT count (*P*: 0.001), and LSM (*P*: 0.03) were shown as variables significantly associated with the HD occurrence, contrarily to diabetes mellitus (*P*: 0.413).

Even in these individuals, the ALBI and the PLT count–based NITs (FIB-4, ALBI-FIB-4, RPR, LSM/PLTr, and ANTICIPATE ± NASH) (all *P* < 0.0001) were predictors of this outcome. However, in this setting of patients, the multivariate analysis highlighted ALBI (*P*: 0.002) and ALBI-FIB-4 (*P*: 0.041) as the variables exclusively associated with the HD, not confirming the other PLT count–based NITs (FIB-4 [*P*: 0.073], RPR [*P*: 0.061], LSM/PLT ratio [*P*: 0.074], and ANTICIPATE ± NASH [*P*: 0.082]) as predicting this outcome, independently from sex, age, BMI, albumin levels, diabetes mellitus, MELD, and NSBB effectiveness (Table 2).

### Predictive Performance of Nits Across the Study Groups

#### Patients Presenting Splenomegaly

**Figure 4:**
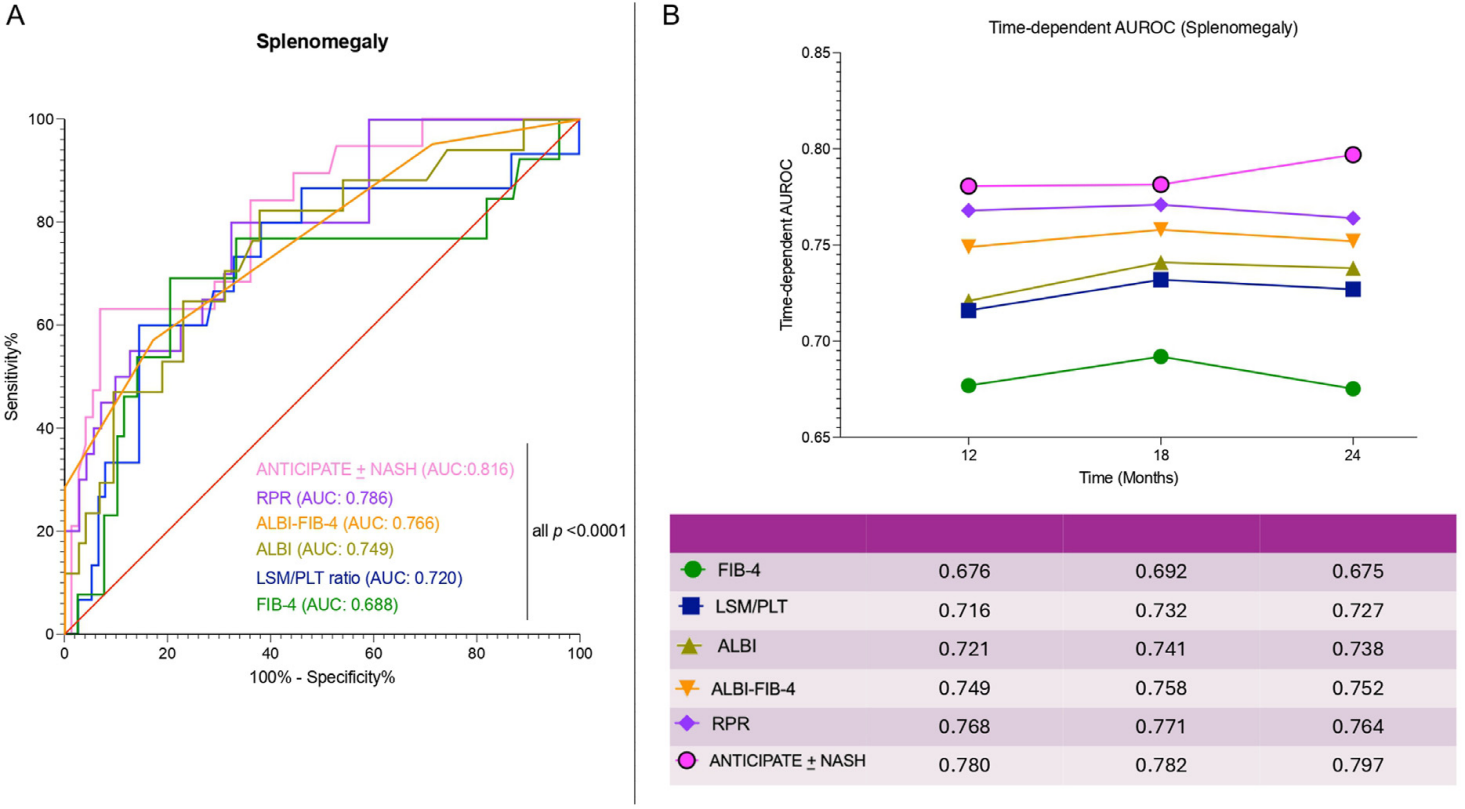
Performance of NITs in predicting hepatic decompensation (SAP). Static (**A**) and dynamic (**B**) performance of the NITs in predicting hepatic decompensation in MASLD-related cACLD patients presenting splenomegaly (SAP). Time-dependent receiving operator curve analysis and DeLong test for curve comparisons. cACLD, compensated advanced chronic liver disease; PLT, Platelet; RDW, red cell distribution width; RPR, red cell distribution width-to-platelet ratio; LSM, liver stiffness measurement; MASLD, metabolic dysfunction–associated steatotic liver disease; SAP, splenomegaly-affected patient; NIT, non-invasive tool; FIB-4, Fibrosis-4; ALBI, Albumin-Bilirubin score; NASH, non-alcoholic steatohepatitis; AUROC, area under the receiver operating characteristic; AUC, area under the curve.

Consistently with the multivariate analysis results, in MASLD-cACLD SAPs presenting CSPH, the ROC analysis evidenced a good accuracy of both ALBI (AUC: 0.749) and PLT count–based NITs (all *P* < 0.0001) in predicting the HD at 3 years (Figure 4, panel A).

In line with this, the time-dependent ROC analysis confirmed the good accuracy in the prediction of HD for FIB-4 (AUC: 0.676), LSM/PLTr (0.716), ALBI (0.721), ALBI-FIB-4 (0.749), RPR (0.768), and ANTICIPATE ± NASH (area under the receiver operating characteristic: 0.780) at 12 months (1 year), with similar results observed at 18 months (1.5 years) and 24 months (2 years) (Figure 4, Panel B). Moreover, comparing the performance of the NITs along the follow-up, the DeLong test evidenced no statistically significant difference between the assessed models at 1, 1.5, and 2 years.

#### Patients Not Presenting Splenomegaly

**Figure 5:**
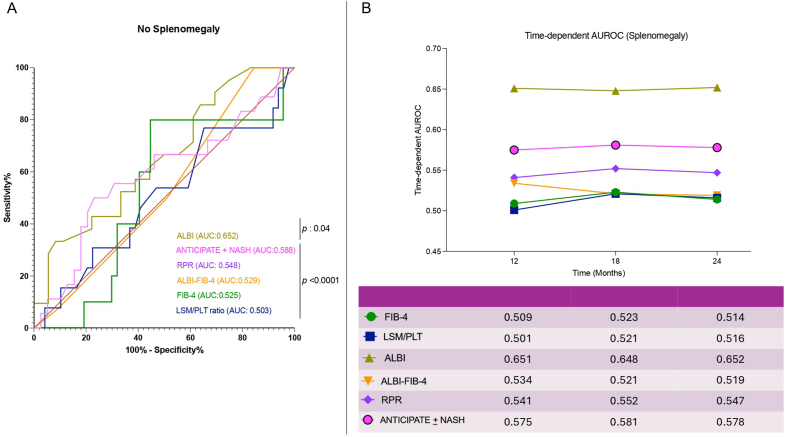
Performance of NITs in predicting hepatic decompensation (NSP). Static (**A**) and dynamic (**B**) performance of the NITs in predicting hepatic decompensation in MASLD-related cACLD patients not presenting splenomegaly (NSP). Time-dependent receiving operator curve analysis and DeLong test for curve comparisons. cACLD, compensated advanced chronic liver disease; PLT, platelet; RDW, red cell distribution width; RPR, red cell distribution width-to-platelet ratio; cACLD, compensated advanced chronic liver disease; LSM, liver stiffness measurement; MASLD, metabolic dysfunction–associated steatotic liver disease; NIT, non-invasive tool; NSP, normal-spleen patient; FIB-4, Fibrosis-4; ALBI, Albumin-Bilirubin score; NASH, non-alcoholic steatohepatitis; AUROC, area under the receiver operating characteristic; AUC, area under the curve.

Contrarily to individuals presenting splenomegaly, in NSPs, poor prognostic accuracy in predicting 3-year decompensation was reported for all PLT count–based NITs (all *P* < 0.0001), with a preserved performance demonstrated exclusively for ALBI (AUC: 0.652, *P*: 0.04) (Figure 5, panel A). Consistently with this, the time-dependent ROC analysis showed a good performance of ALBI (AUC: 0.651), in contrast with poor predictive accuracy for HD for all the PLT count–based NITs at 12 months (1 year) (FIB-4 [AUC: 0.509], LSM/PLTr [0.501], ALBI-FIB-4 [0.534], RPR [0.541], and ANTICIPATE ± NASH [0.575]), with similar results observed at 18 months (1.5 years) and 24 months (2 years) (Figure 5, panel B).

Relevantly, comparing the NIT's performance along the follow-up, the DeLong test revealed no significant differences between the PLT count–based NITs, as well as the superiority of ALBI in comparison to all the PLT count–based NITs at 1, 1.5, and 2 years (Table 3).

**Table 3:** Comparison of ALBI vs Platelet Count–Based NITs Dynamic Predictive Performance (NSPs and ASP Individuals) in Predicting Hepatic Decompensation.

NSP MASLD-cACLD-CSPH patients							
	ALBI (A)	FIB-4 (B)	LSM-PLTr (C)	ALBI-FIB-4 (D)	RPR (E)	ANTICIPATE ± NASH (F)	***P*** value^a^
12 months	0.651	0.509	0.501	0.534	0.541	0.575	A vs B: **<0.0001**
							A vs C:**<0.0001**
							A vs D: **0.001**
							A vs E: **0.002**
							A vs F: **0.003**
18 months	0.648	0.523	0.521	0.521	0.552	0.581	A vs B: **0.001**
							A vs C: **0.001**
							A vs D: **0.001**
							A vs E: **0.002**
							A vs F: **0.02**
24 months	0.652	0.514	0.516	0.519	0.547	0.578	A vs B: **<0.0001**
							A vs C:**<0.0001**
							A vs D: **0.001**
							A vs E: **0.002**
							A vs F: **0.003**

ASP MASLD-cACLD-CSPH patients							
	ALBI (A)	FIB-4 (B)	LSM-PLTr (C)	ALBI-FIB-4 (D)	RPR (E)	ANTICIPATE ± NASH (F)	*P* value^a^
12 months	0.649	0.531	0.509	0.537	0.538	0.571	A vs B: **<0.0001**
							A vs C: **<0.0001**
							A vs D: **0.001**
							A vs E: **0.002**
							A vs F: **0.003**
18 months	0.645	0.527	0.523	0.525	0.547	0.584	A vs B: **0.001**
							A vs C: **0.001**
							A vs D: **0.001**
							A vs E: **0.002**
							A vs F: **0.02**
24 months	0.650	0.516	0.512	0.516	0.541	0.575	A vs B: **<0.0001**
							A vs C: **<0.0001**
							A vs D: **0.001**
							A vs E: **0.002**
							A vs F: **0.003**

Statistically significant differences (*P* < 0.05) are reported in bold. B vs C, B vs D, B vs E, B vs F, C vs D, C vs E, C vs F, D vs E, D vs F, and E vs F were not statistically significant differences.

ASP, asplenic; NSP, normal spleen patients; CSPH, clinically significant portal hypertension; cACLD, compensated advanced chronic liver disease; PLT, platelet; RPR, red cell distribution width-to-platelet ratio; LSM, liver stiffness measurement; LSM/PLTr, liver stiffness measurement-to-platelet ratio; FIB-4, Fibrosis-4; ALBI, Albumin-Bilirubin score; MASLD, metabolic dysfunction–associated steatotic liver disease; NIT, non-invasive tool; NSP, normal-spleen patient; NASH, non-alcoholic steatohepatitis.

a DeLong test comparison.

#### ASP Patients

Finally, similarly to NSPs, in ASP individuals, ALBI showed a good performance in predicting the HD at 3 years (AUC: 0.625; *P*: 0.03), whereas all PLT count–based NITs exhibited poor predictive accuracy ([FIB-4, AUC: 0.563; LSM/PLT ratio, AUC: 0.523; ALBI-FIB-4, AUC: 0.595; RPR, AUC: 0.598; ANTICIPATE ± NASH, AUC: 0.601] [all *P* < 0.0001]).

Consistently, with the findings reported for MASLD-cACLD NSP, comparing the performance of NITs during the follow-up, no significant differences between the PLT count-based NITs were revealed, whereas the superiority of ALBI in comparison to all these tools emerged at 1, 1.5, and 2 years (Table 3).

## DISCUSSION

PLT count–enclosing NITs have been recently shown to provide prognostic information comparable to the HVPG in predicting the short-term risk of decompensation in cACLD.^12^ Anyway, in the previous studies exploring the applications of PLT count–based NITs, patients were not stratified according to spleen size,^5^, ^6^, ^7^, ^8^, ^9^^,^^11^^,^^12^ which emerged as a crucial feature impacting this outcome in other research, both in terms of volume and area.^37^^,^^38^ In particular, Yu *et al.* adopted an artificial intelligence (AI)-based (deep learning) segmentation network generating the spleen volume to elaborate a model that outperformed the serum-based ones in predicting decompensation in cACLD, providing a user-friendly method in diverse healthcare settings where the HVPG and stiffness measurement are unavailable.^37^ More recently, preliminary observations suggested the impact of the spleen area in influencing the performance of serum-based models in predicting decompensation in MASLD cirrhosis.^38^

The MASLD-cACLD configured a priority setting for investigating this unsolved question, given also the alarming epidemiological projections defining metabolic dysfunction–associated hepatopathy as the primary cause of chronic liver damage worldwide,^19^^,^^20^ the evidence of obese NASH constituting a “gray zone” in the Baveno VII consensus-proposed algorithm to determine CSPH non-invasively,^31^ and the already demonstrated close relationship between spleen dimensions and fat-free mass in obese patients.^21^

In our cohort of MASLD-CSPH–affected patients, the prevalence of splenomegaly (I: SAPs [61.48%] and II: NSPs [38.52%]) reflected the epidemiological data reported in the literature.^16^

To avoid the measurement bias affecting previous research evaluating the spleen size exclusively by bipolar diameter with a non-negligible risk of operator-accuracy dependence,^4^^,^^10^^,^^39^ we specifically used an AI-based tool able to produce an accurate measurement of the spleen size, repeating all the procedures in duplicate. In addition, the prevalence of splenomegaly was not significantly different among males and females (*P*: 0.3043) in both group I and II, as well as no statistically significant differences were found between the groups for BMI and BSA.

A plethora of evidence suggests systemic inflammation, synergically with CSPH, represents the key driver of liver disease worsening and decompensation in ACLD.^1^^,^^40^

Anyway, no statistically significant baseline differences emerged between the groups, neither regarding hepatic functionality (CP and MELD), liver disease progression (LSM), and systemic inflammation (CRP) nor concerning CSPH severity. Moreover, considering the well-known simultaneous influence of systemic inflammation on hematopoiesis (“reactive platelet disease”),^11^^,^^41^^,^^42^ the baseline results revealed no differences in the comparison of pathophysiologically linked variables (PLT count, CRP, and RDW-SD) among the groups, reinforcing this assumption.

Concerning the PLT count relationship with the two spleen size measurements, a significantly negative correlation (R: −0.751) with the spleen diameter, as well as an even more relevant inverse correlation with the spleen area (R: −0.981), was reported exclusively in SAPs. These results confirmed the predominant impact of splenomegaly-related hypersplenism on PLT count, authorizing the investigation of decompensation-predicting factors in the three study groups.

Concerning decompensation, following the clinical research trends on the topic available in literature during the realization of the study,^43^^,^^44^ besides the “traditional” LRDEs (ascites, hepatic encephalopathy, and bleeding), even progressive jaundice in non-cholestatic cirrhosis, any grade (≥ grade 1) of hepatic encephalopathy, as well as any type of acute bacterial infection requiring hospitalization, defined the transition to decompensated ACLD in our research, according to the recently proposed novel definition of HD in cirrhosis with relative patterns.^1^^,^^40^

In light of this modern solid evidence,^1^^,^^40^ the recording of “untraditional” liver-related events appeared conceivable and reasonable without the risk of overestimating HD. Anyway, this last possibility appears even more remote, considering jaundice represented only a limited portion of LRDEs occurring in our study (consistent with the data of other research), and, despite the inclusion in the recording proposals, no patients developed acute bacterial infections requiring hospitalizations as a first decompensating event during the follow-up (taking that in mind, according to the experimental design, the first LRDE occurrence represented the end of follow-up).

In SAPs, the multivariate analysis revealed ALBI and the PLT count–based NITs as the exclusive variables significantly predicting decompensation, not confirming the significance of spleen area. In NSPs, PLT count–based NITs showed a reduced influence (*i.e.*, a relatively lower aSHR) on the outcome compared to SAPs, and unlike this, the spleen area was confirmed as inversely associated (aSHR: 0.870), also emerging as the variable more significantly impacting decompensation. Finally, in ASP, ALBI, and ALBI-FIB-4 were the variables exclusively associated with HD.

From a practical point of view, these results suggest the spleen area can dramatically influence the decompensation in NSPs and, simultaneously, even considering the previously reported relationship with the PLT count, impact on the LRDEs’ predictive association of the PLT count–based NITs. As a *deus ex machina*, the ASP findings reinforce these associations. Consistent with this, in SAPs, both ALBI and PLT count–based NITs showed good prognostic accuracy in predicting HD at 3 years.

Differently, in NSPs, the ALBI score was the only tool to maintain a preserved performance (AUC: 0.652), while all PLT count–based NITs showed poor prognostic accuracy in predicting 3-year decompensation. In this group, the superiority of ALBI in comparison to all the PLT count–based NITs was evidenced at 1, 1.5, and 2 years, reporting similar findings also in ASP individuals.

Our study presents limitations: first, this is a monocentric observation; second, although its evaluation was not essential for the main research outcome, HVPG measurement has not been included as the reference standard comparator or as the standard tool to assess NSBB effectiveness.

Although the HVPG represents the gold standard to define CSPH, given the non-routine availability of this method, alternative undirect methods are adopted to define CSPH in clinical practice.^3^^,^^9^^,^^31^ As revealed by solid evidence, the main clinical consequence of portal hypertension (i.e., the formation of esophageal varices) occurs only in the presence of HVPG values of at least 10–12 mmHg; consequently, all patients presenting esophageal varices have HVPG values >10 mmHg and thus CSPH.^24^^,^^36^ Moreover, patients with CSPH-related endoscopic signs present a significant risk of decompensation.^34^ This solid evidence guided our choice to consider CSPH exclusively in the presence of endoscopic varices in patients receiving esophagogastroduodenoscopy (EGDS) according to the varix-related screening endoscopy guidelines.^25^^,^^31^^,^^34^ Certainly, it is plausible that a limited part of cACLD patients with radiological signs of portal hypertension not receiving EGDS (and/or receiving—for other reasons—an EGDS revealing varices) could present misdiagnosed CSPH without performing HVPG.

Anyway, this represents a very limited setting constituting an unsolved grey zone,^25^^,^^31^^,^^34^ as well as a generalized hepatologic/scientific community research unmet need,^31^ certainly not limited to our study and not constituting an objective of the present research.

Even the VITRO^9^ was a PLT count–incorporating NIT not included in this research since for a large timeframe, the VWF assessment was not accessible at our department for a great proportion of patients due also to Severe Acute Respiratory Syndrome-CoV2-pandemic–related logistic difficulties.

The emerging findings should be intended as preliminary encouraging results opening, in a pioneering way, the doors to further studies on larger populations investigating the reproducibility of these features even in etiologies other than MASLD. Looking to future perspectives, the assessment of the spleen area as a previously unexplored, easy, and objective parameter to assess to stratify patients with and without splenomegaly takes on a potentially key role in the construction of reliable tailored NITs as a valid alternative to the more expensive and less accessible spleen stiffness measurement, as recently proposed in other models such as the novel designed “Non-Invasive CSPH Estimated Risk” (“NICER”).^45^

## Credit authorship contribution statement

M.R. and M.D.: guarantor of the article, conceptualization, methodology, investigation, and writing the original draft; F.D.N. and C.N.: conceptualization, methodology, formal analysis, investigation, and writing the original draft. P.V., S.O., and M.N.: investigation, resources, data curation, and visualization; A.F.: conceptualization, data curation, and supervision. All authors approved the final version of the manuscript.

## Ethics approval and consent to participate

This study follows the Declaration of Helsinki (1975) and has been approved by the ethical committee of the University of Campania Luigi Vanvitelli in Naples (prot n. 0018123/2015). All study participants, or their legal guardians, provided informed written consent before study enrollment.

## Availability of data and materials

The data that support the findings of this study are available from the corresponding author upon reasonable request.

## Funding

This research received no financial support.

## Declaration of competing interest

All the authors declare no conflict of interest and no competing interests.
