# Supplementary material for: Spleen Area Affects the Performance of the Platelet Count–Based Non-invasive Tools in Predicting First Hepatic Decompensation in Metabolic Dysfunction–Associated Steatotic Liver Disease Cirrhosis
Source: J Clin Exp Hepatol. 2025 May 27;15(6):102596. doi: 10.1016/j.jceh.2025.102596 (PMC12209911; doi:10.1016/j.jceh.2025.102596)
Supplement: Multimedia component 2 [file mmc2.pdf]

Supplementary File 2- Methods: LSM, Spleen Size, NITs assessment (bullet point)

- ***Liver Stiffness Measurement***

  LSM was obtained by using FibroScan® [version 502 (Echosens, Paris, France)] with M and XL probes. We used the XL probe when the ultrasound-measured distance between the skin and the liver capsule was greater than 2.5 cm and/or when the patient's BMI was >30. FibroScan® was performed by an expert physician obtaining 10 acceptable measurements, with the maximum number of attempts set at 20. The criteria proposed by *Boursier et al.* defined measurement as "very reliable" (IQR/M ≤ 0.1), "reliable" (0.1 < IQR/M ≤ 0.3 or IQR/M > 0.3 with LS median < 7.1 kilopascal), or "poorly reliable" (IQR/M > 0.3 with LS median ≥ 7.1 kPa) [1,2].

- ***Spleen Size Measurement***

  The US examination was conducted with a GE Logiq E10™ instrument using a "C1-6-D" XDclear Convex 1-6 MHz probe supported by the artificial intelligence (AI)-based tool "A to A Digital Platform GE™ and a complete evaluation of the liver and the spleen-portal axis was performed independently by two expert physicians for each patient. The spleen was evaluated with a supine decubitus patient via the intercostal window to acquire the broadest possible scan including the hilum and both measure the bipolar Spleen Diameter (expressed in cm) and the Spleen Area (cm^2) [3]. Concerning this, specific equipment ("Anatomical Assistant") permitted the dynamic prolonged record of sequential spleen-focused windows in deep inspiration, as well as to freeze, and thus automatically select the best possible scan (BPS). After obtaining the BPS, the machine was automatically able to track the bipolar Spleen Diameter and the organ's perimeter, ultimately producing an accurate measurement of the Spleen Area ("Measure Assistant"). During the entire procedure, the operator (physician 1) was always surveilled, and all the actions were constantly externally reviewed, via projecting the image on a portable device (phone or tablet) ("Remote assistant"), by a second expert physician (physician 2) (**Supplementary Figure 1**). Moreover, all the procedures were made in duplicate by each physician, and the mean of the mean of physician 1 and physician 2 measurements were conclusively recorded.

Supplementary File 2- Methods: LSM, Spleen Size, NITs assessment (bullet point)

- ***Non-invasive tools predicting hepatic decompensation***

To noninvasively determine the risk of hepatic decompensation, the determination of ALBI [4] and the calculation of the following PLT count-based NITs [including FIB-4 [5], ALBI-FIB-4 [6], LSM/PLTr [7], RPR [8], and the estimation of individual CSPH risk according to the ANTICIPATE, or in obese patients, the ANTICIPATE-NASH (ANTICIPATE ± NASH) model [9]] were performed. In detail, the ALBI score was calculated as ALBI = [-0.085 × (albumin) (g/L) + 0.66 × log10 (total bilirubin) (mmol/L)] [10].

FIB-4 score, a non-invasive estimation of liver fibrosis, was calculated by using the originally described formula [5]: Age (years) × AST/PLT count (10^3/mL) × ALT½. FIB-4 categories were: (1) Low risk for advanced fibrosis (AF) (< 1.45); (2) high risk for AF (> 3.25); or (3) indeterminate (1.45-3.25) [5].

The combined score ALBI-FIB-4 stratified patients as follows: I group of risk (ALBI ≤ -2.60 and FIB-4 ≤ 3.25); II group of risk (ALBI ≥ -2.60 and FIB-4 ≤ 3.25); III group of risk (ALBI ≤ -2.60 and FIB-4 ≥ 3.25); IV group of risk (ALBI ≥ -2.60 and FIB-4 ≥ 3.25) [6].

The LSM/PLTr was calculated by dividing LSM (kPa) by PLT count (u/microL) [7], whereas the RPR was determined by using the formula: RDW-SD/PLT count (u/microL) [8].

The ANTICIPATE, or in obese patients, the ANTICIPATE-NASH (ANTICIPATE ± NASH) model was calculated according to the published formulas [11]. Results were all confirmed and the probability was estimated by using the online calculator https://www.bcn-liverhuvh.com/resources.

References

[1] Eddowes PJ, Sasso M, Allison M, et al. Accuracy of FibroScan Controlled Attenuation Parameter and Liver Stiffness Measurement in Assessing Steatosis and Fibrosis in Patients With Nonalcoholic Fatty Liver Disease. Gastroenterology 2019;156:1717–30. https://doi.org/10.1053/j.gastro.2019.01.042.

[2] Boursier J, Zarski J-P, De Ledinghen V, et al. Determination of reliability criteria for liver stiffness evaluation by transient elastography. Hepatology 2013;57:1182–91. https://doi.org/10.1002/hep.25993.

[3] Giuffrè M, Macor D, Masutti F, et al. Evaluation of spleen stiffness in healthy volunteers using point shear wave elastography. Ann Hepatol 2019;18:736–41. https://doi.org/10.1016/j.aohep.2019.03.004.

[4] Toyoda H, Johnson PJ. The ALBI score: From liver function in patients with HCC to a general measure of liver function. JHEP Rep 2022;4:100557. https://doi.org/10.1016/j.jhepr.2022.100557.

[5] Sterling RK, Lissen E, Clumeck N, et al. Development of a simple noninvasive index to predict significant fibrosis in patients with HIV/HCV coinfection. Hepatology 2006;43:1317–25. https://doi.org/10.1002/hep.21178.

[6] Liao R, Li D-W, Du C-Y, Li M. Combined Preoperative ALBI and FIB-4 Is Associated with Recurrence of Hepatocellular Carcinoma After Curative Hepatectomy. J Gastrointest Surg 2018;22:1679–87. https://doi.org/10.1007/s11605-018-3810-1.

[7] Berzigotti A, Seijo S, Arena U, et al. Elastography, spleen size, and platelet count identify portal hypertension in patients with compensated cirrhosis. Gastroenterology 2013;144:102-111.e1. https://doi.org/10.1053/j.gastro.2012.10.001.

[8] Dallio M, Romeo M, Vaia P, et al. Red cell distribution width/platelet ratio estimates the 3-year risk of decompensation in Metabolic Dysfunction-Associated Steatotic Liver Disease-induced cirrhosis. World J Gastroenterol 2024;30:685–704. https://doi.org/10.3748/wjg.v30.i7.685.

[9] Pons M, Rivera-Esteban J, Ma MM, et al. Point-of-Care Noninvasive Prediction of Liver-Related Events in Patients With Nonalcoholic Fatty Liver Disease. Clin Gastroenterol Hepatol 2023:S1542-3565(23)00626-2. https://doi.org/10.1016/j.cgh.2023.08.004.

[10] Demirtas CO, D'Alessio A, Rimassa L, Sharma R, Pinato DJ. ALBI grade: Evidence for an improved model for liver functional estimation in patients with hepatocellular carcinoma. JHEP Rep 2021;3:100347. https://doi.org/10.1016/j.jhepr.2021.100347.

[11] Rabiee A, Deng Y, Ciarleglio M, et al. Noninvasive predictors of clinically significant portal hypertension in NASH cirrhosis: Validation of ANTICIPATE models and development of a lab-based model. Hepatol Commun 2022;6:3324–34. https://doi.org/10.1002/hep4.2091.
