# Supplementary figures and images for: Spleen Area Affects the Performance of the Platelet Count–Based Non-invasive Tools in Predicting First Hepatic Decompensation in Metabolic Dysfunction–Associated Steatotic Liver Disease Cirrhosis
Source: J Clin Exp Hepatol. 2025 May 27;15(6):102596. doi: 10.1016/j.jceh.2025.102596 (PMC12209911; doi:10.1016/j.jceh.2025.102596)

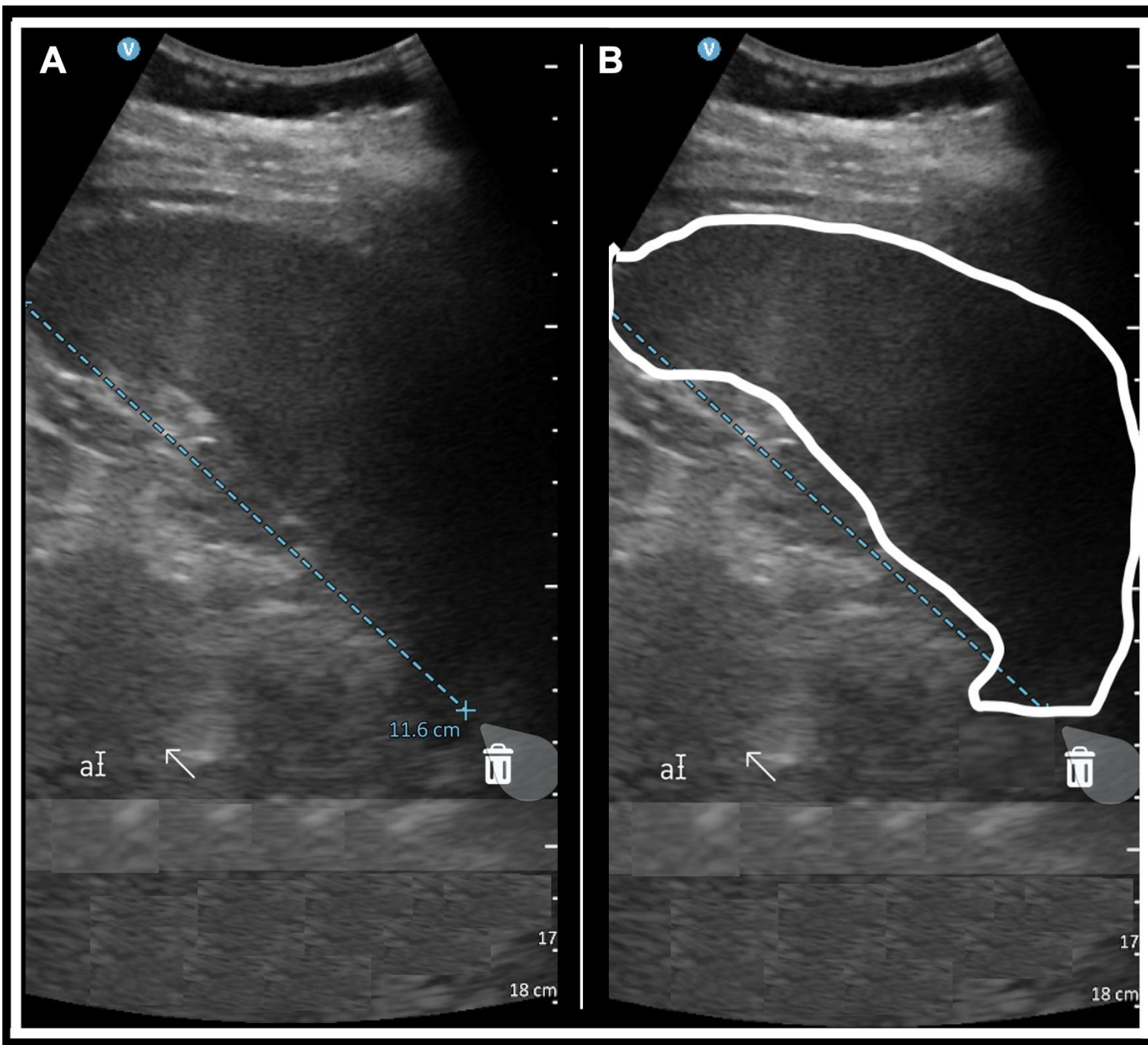

Supplement: Multimedia component 3 [file mmc3.pdf]

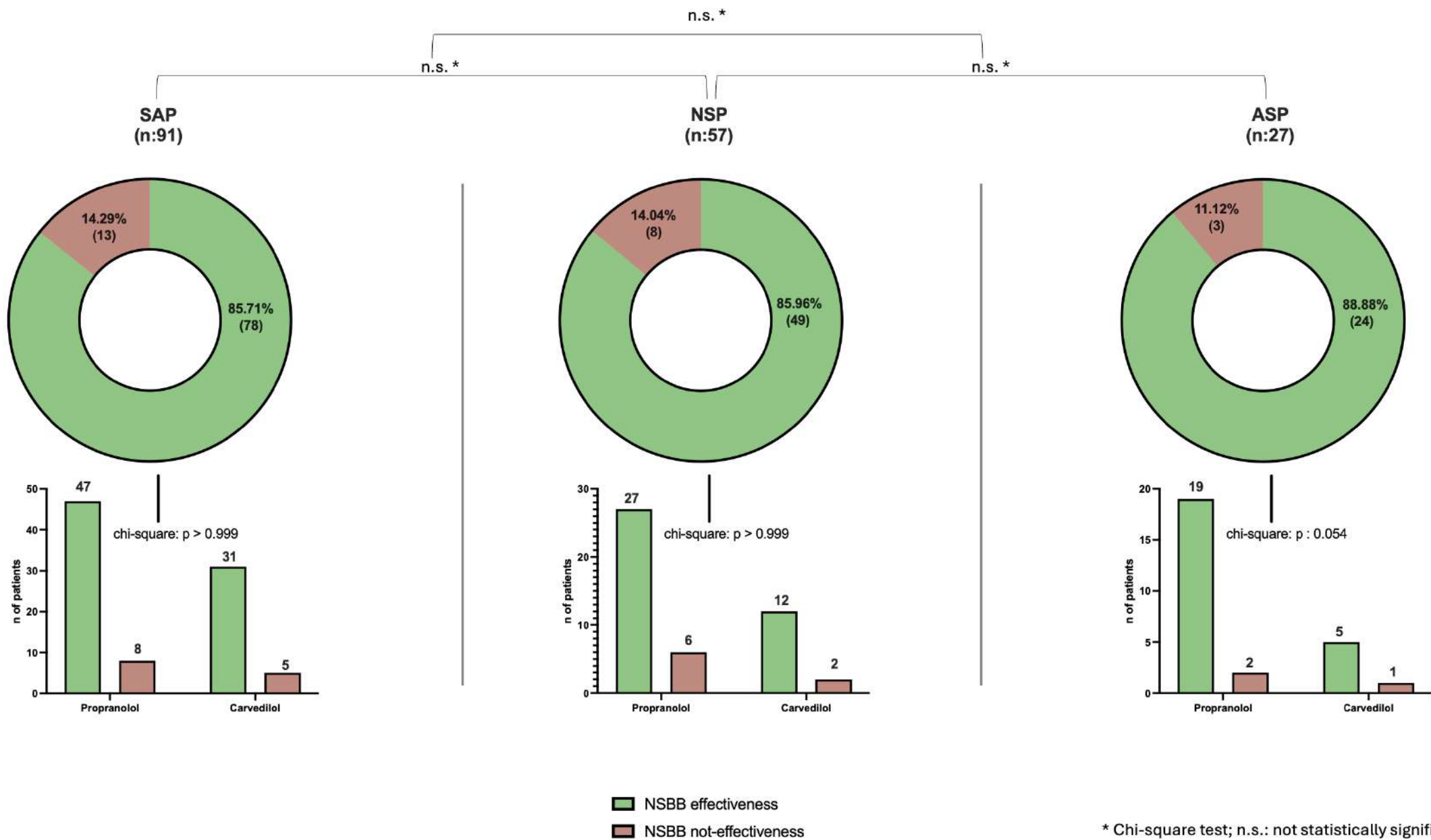

\* Chi-square test; n.s.: not statistically significant

Supplement: Multimedia component 5 [file mmc5.pdf]
