# Supplementary material for: Spleen Area Affects the Performance of the Platelet Count–Based Non-invasive Tools in Predicting First Hepatic Decompensation in Metabolic Dysfunction–Associated Steatotic Liver Disease Cirrhosis
Source: J Clin Exp Hepatol. 2025 May 27;15(6):102596. doi: 10.1016/j.jceh.2025.102596 (PMC12209911; doi:10.1016/j.jceh.2025.102596)
Supplement: Multimedia component 4 [file mmc4.pdf]

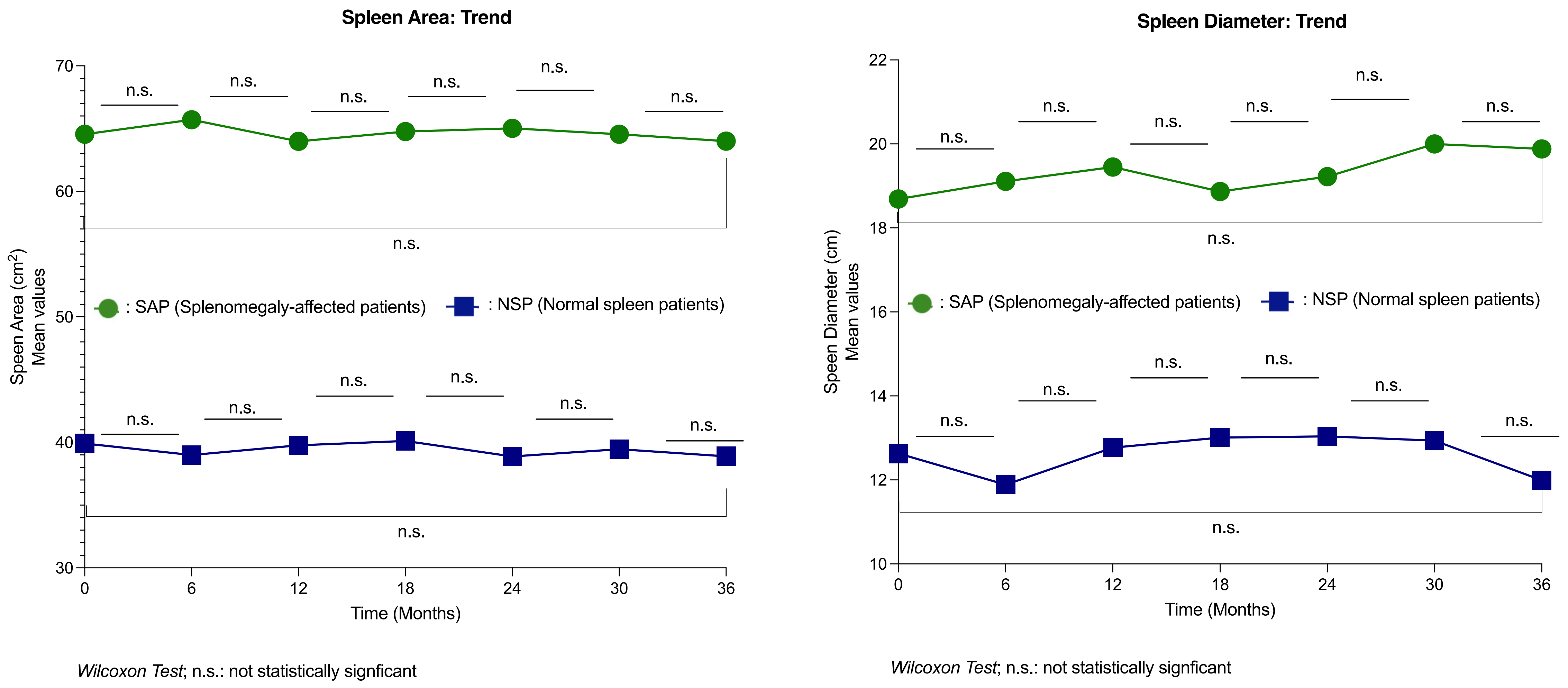
Spleen Area: Trend
Speen Area (cm^2)
Mean values
70
60
50
40
30
n.s.
: SAP (Splenomegaly-affected patients)
: NSP (Normal spleen patients)
0
6
12
18
24
30
36
Time (Months)
Wilcoxon Test; n.s.: not statistically signficant
Spleen Diameter: Trend
Speen Diameter (cm)
Mean values
22
20
18
16
14
12
10
n.s.
: SAP (Splenomegaly-affected patients)
: NSP (Normal spleen patients)
0
6
12
18
24
30
36
Time (Months)
Wilcoxon Test; n.s.: not statistically signficant
