# Supplementary material for: Spleen Area Affects the Performance of the Platelet Count–Based Non-invasive Tools in Predicting First Hepatic Decompensation in Metabolic Dysfunction–Associated Steatotic Liver Disease Cirrhosis
Source: J Clin Exp Hepatol. 2025 May 27;15(6):102596. doi: 10.1016/j.jceh.2025.102596 (PMC12209911; doi:10.1016/j.jceh.2025.102596)
Supplement: Multimedia component 6 [file mmc6.pdf]

Supplementary Table 1. Frequency distribution of LRDEs' types during the follow-up period in the three population groups.

	**SAP (n:91*) (I)**	**NSP (n:57*) (II)**	**ASP (n:27*) (III)**	**p-value**
**Type of LRDEs occurred during the follow-up period**	**Total LRDEs: 17**	**Total LRDEs: 11**	**Total LRDEs: 6**	
(A) Slow/ grade 1 ascites formation (n and %)	6 (35.4%)	4 (36.3%)	2 (33.3%)	I vs II: n.s.<br>II vs III: n.s.<br>I vs III: n.s.
(B) Mild (grade 1/2) Hepatic Encephalopathy (n and %)	3 (17.6%)	2 (18.2%)	1 (16.7%)	I vs II: n.s.<br>II vs III: n.s.<br>I vs III: n.s.
(C) Jaundice in non-cholestatic cirrhosis (n and %)	3 (17.6%)	2 (18.2%)	1 (16.7%)	I vs II: n.s.<br>II vs III: n.s.<br>I vs III: n.s.
(A) + (B) (n and %) / (A) + (C) (n)	5 (29.4%)	3 (27.3%)	2 (33.3%)	I vs II: n.s.<br>II vs III: n.s.<br>I vs III: n.s.

Chi-square test analysis. Statistically significant differences (p<0.05); not statistically significant; LRDE: Liver-related decompensation event; SAP: splenomegaly-affected patients; NSP: Normal spleen patients; ASP: Asplenic individuals. *16 (9.14%) patients were lost and did not complete follow-ups [8 (8.79%) SAP, 5 (8.77%) NSP, and 3 (11.11%) ASP].
